# Natural compound-induced downregulation of antimicrobial resistance and biofilm-linked genes in wastewater *Aeromonas* species

**DOI:** 10.3389/fcimb.2024.1456700

**Published:** 2024-10-14

**Authors:** Khristina G. Judan Cruz, Okamoto Takumi, Kenneth A. Bongulto, Emmanuel E. Gandalera, Ngure Kagia, Kozo Watanabe

**Affiliations:** ^1^ Center for Marine Environmental Studies (CMES), Ehime University, Matsuyama, Ehime, Japan; ^2^ Department of Biological Sciences, College of Science, Central Luzon State University, Science City of Muñoz, Nueva Ecija, Philippines

**Keywords:** ARGS, antimicrobial resistance, biofilm, natural compounds, *Aeromonas*, wastewater

## Abstract

Addressing the global antimicrobial resistance (AMR) crisis requires a multifaceted innovative approach to mitigate impacts on public health, healthcare and economic systems. In the complex evolution of AMR, biofilms and the acquisition of antimicrobial resistance genes (ARGs) play a pivotal role. *Aeromonas* is a major AMR player that often forms biofilm, harbors ARGs and is frequently detected in wastewater. Existing wastewater treatment plants (WWTPs) do not have the capacity to totally eliminate antimicrobial-resistant bacteria favoring the evolution of ARGs in wastewater. Besides facilitating the emergence of AMR, biofilms contribute significantly to biofouling process within the activated sludge of WWTP bioreactors. This paper presents the inhibition of biofilm formation, the expression of biofilm-linked genes and ARGs by phytochemicals andrographolide, docosanol, lanosterol, quercetin, rutin and thymohydroquinone. *Aeromonas* species were isolated and purified from activated sludge samples. The ARGs were detected in the isolated *Aeromonas s*pecies through PCR. *Aeromonas* biofilms were quantified following the application of biocompounds through the microtiter plate assay. qPCR analyses of related genes were done for confirmation. Findings showed that the natural compounds inhibited the formation of biofilms and reduced the expression of genes linked to biofilm production as well as ARGs in wastewater *Aeromonas*. This indicates the efficacy of these compounds in targeting and controlling both ARGs and biofilm formation, highlighting their potential as innovative solutions for combating antimicrobial resistance and biofouling.

## 1 Introduction

Wastewater treatment plants (WWTPs) and wastewater are critical transition points for the emergence of antimicrobial-resistant bacteria (ARB) and antimicrobial resistance genes (ARG) (Skwor et al., 2020) following the release of residual antibiotics not fully metabolized after therapeutic use. Since existing WWTPs do not have the capacity to totally eliminate pollutants specifically ARB (Shrout and Nerenberg, 2012; Uluseker et al., 2021; Perveen et al., 2023), this creates a favorable environment for the expansion of antimicrobial resistance. Among the diverse bacterial species found in wastewater, *Aeromonas* species are known to harbor a wide range of ARGs and virulence factors, making them ideal model organisms for monitoring AMR and studying the mechanisms of resistance development (Usui et al., 2016; Skwor et al., 2020). *Aeromonas* genomes harboring ARGs have increased considerably (Roh and Kannimuthu, 2023) and highlights their potential as a global public health risk (Piotrowska et al., 2017; Skwor et al., 2020).

Aside from facilitating the acquisition and exchange of ARGs, *Aeromonas* is also known for forming biofilms in water systems (Talagrand-Reboul et al., 2017). Biofilm architecture ensures stability and barrier (Hall-Stoodley et al., 2004; Rather et al., 2021) and makes biofilms particularly resistant to antibiotics. Studies have shown a substantial increase in antibiotic resistance in biofilm-associated bacteria compared to their planktonic forms, as well as a high frequency of ARGs (Chen et al., 2020; Michaelis and Grohmann, 2023). *Aeromonas*, akin to other bacteria, can induce biofouling in WWTP activated sludge bioreactors by forming biofilms on surfaces, filters, pumps, and membranes (Taiswa et al., 2024). This compromises the flow of water through the system, leading to decreased pressure and increased energy consumption, resulting in reduced efficiency of filters, membranes and flux rate and surge in maintenance costs.

ARGs and biofilm play a key part in the emergence of antimicrobial resistance and has been linked to most persistent infections. Strategies and emerging therapeutic options for antipathogenesis in MDR bacteria are limited, leading to higher risks of AMR development, increased morbidity and mortality rates and consequently, significant economic burdens. As antibiotics become less effective while more multi-drug resistant (MDR) pathogens emerge and spread globally, research efforts and new antimicrobials are urgently needed to produce novel, effective strategies not only for the public health but also to mitigate effects on significant economic losses (WHO, 2023). Targeting two mechanisms at the same time, such as ARGs and biofilm is a practical alternative to antibiotics use and display high potential in efficiently managing bacterial pathogenicity and antimicrobial resistance.

Natural products are now being extensively explored for their ability to interfere with ARGs and biofilm formation in bacteria. Plants have evolved intricate defense mechanisms, including the production of a wide array of secondary metabolites, many of which exhibit antimicrobial properties and play an integral part in the plant’s immune system (Algburi et al., 2017). The exploration of natural products for their potential to interfere with ARGs and biofilms is an emerging area of antimicrobial research. Several natural products have been shown to modulate or inhibit biofilm formation and downregulate ARG and biofilm-linked genes. The enormous diversity of natural products provides a vast reservoir of antimicrobial compounds that may contribute to the development of novel antimicrobial drugs to combat resistance mechanisms in bacteria.

The role in pathogenesis and increasing incidences of persistent infections influenced by ARGs and biofilm formation makes these important targets in the development of antimicrobials for *Aeromonas* sp. while avoiding the development of AMR driven by antibiotic over-use and mis-use. The capacity of natural compounds to influence biofilm formation and the expression of biofilm-linked genes and ARGs offers a plausible approach to addressing AMR in *Aeromonas*. Hence, this paper explores the action of six plant-derived compounds to inhibit the formation of biofilm and downregulate the expression of biofilm-linked genes and ARGs in 6 wastewater *Aeromonas* species.

## 2 Materials and methods

### 2.1 Isolation and purification of bacteria

*Aeromonas* species were isolated from wastewater activated sludge collected from bioreactors in 2 wastewater treatment facilities in Japan. A total of 3 liters of water were collected. The water samples were immediately filtered using a 50 µm mesh, stored in ice and processed within 3 hours. Appropriately diluted samples were plated onto Mueller-Hinton Agar plates and incubated at 37°C for 24 h. Colonies were picked on the plates and inoculated to 20 ml Luria-Bertani (LB) Broth and incubated for 24 h at 37 ^0^C. Pure bacterial cultures were obtained after successive subculture of colonies in LB plates.

### 2.2 Identification of bacteria

Sixty isolates were subjected to DNA extraction and 16srRNA PCR amplification to identify the bacterial species present in the collected wastewater activated sludge. Genomic DNA was extracted using the DNeasy kit (Qiagen) following the manufacturer’s protocol. In 2ml tubes, 1.75 ml of bacterial culture was centrifuged at 20,000 x g for 5 minutes. The resulting supernatant was decanted. 180 µl of lysis buffer was added to the tube, vortexed and incubated at 37°C for 30 minutes. 25 μl of proteinase K and 200 ul of Buffer AL were added to the tube, vortexed and incubated at 56°C for 30 minutes. 200 µl 95% ethanol was added to the sample and vortexed. This was transferred to the DNeasy spin column placed in a 2 ml collection tube centrifuged at ≥6000 x g (8000 rpm) for 1 minute. The flow-through and collection tube was discarded. The spin column was placed in a new 2 ml collection tube. 500 μl Buffer AW1 was added and centrifuged for 1 min at ≥6000 x g. The flow-through and collection tube was discarded. The spin column was placed in a new 2 ml collection tube. 500 μl Buffer AW2 was added and centrifuged for 3 min at 20,000 x g (14,000 rpm). The flow-through and collection tube was discarded. The spin column was transferred to a new 1.5 ml or 2 ml microcentrifuge tube. To elute the DNA, 100 μl Buffer AE was added to the center of the spin column membrane, incubated for 1 min at room temperature (15–25°C) and centrifuge for 1 min at ≥6000 x g. The DNA was stored at -20°C until use. 16S PCR amplification was performed to accurately identify the bacteria. PCR amplification was done in a mix containing molecular-grade water, 10X Taq buffer, DMSO, MgCl_2_, dNTPs, primers, Taq polymerase and DNA samples. Primers used are the following: 16S 341F 5’-CCTACGGGAGGCAGCAG-3’ and 16S 907R 5’-CCGTCAATTCMTTTRAGTTT-3’ (Lane, 1991). PCR conditions include initial denaturation at 94 °C for 3 minutes followed by 30 cycles of 94 °C, 52 °C and 72 °C; and a final extension of 72°C for 5 minutes. The PCR products were visualized using 1.5% agarose gel and sequenced. Sequences were run in NCBI BLAST for the identification of *Aeromonas* species.

### 2.3 Detection of ARGs

For the detection of ARGs, PCR amplification was done using specific primers (**Table 1**). Primers generated in this study were designed using the Primer3 program (Untergasser et al., 2012). The reaction mix included molecular-grade water, 10X Taq buffer, DMSO, MgCl_2_, dNTPs, primers, Taq polymerase and DNA. PCR conditions include initial denaturation at 94 °C for 5 minutes followed by 30 cycles of denaturation 94°C, annealing temperature range of 50 °C – 60 °C and extension of 72 °C; and a final extension of 72 °C for 5 minutes. The PCR products were visualized using 1.5% agarose gel and sequenced. Known DNA samples positive for the ARGs were ran alongside the samples of this study on agarose gel electrophoresis. The ARG sequences were checked on ResFinder 4.5.0 - Center for Genomic Epidemiology (CGE) (Clausen et al., 2018; Bortolaia et al., 2020). The selection of the primer pairs facilitated the identification of prevalent and clinically significant ARGs, allowing an initial assessment of compound effects.

**Table 1:** Gene primer sequences and references used for the detection of ARGs.

ARGs	Primer sequences	References
TetS F	GAAAGCTTACTATACAGTAGC	Aminov et al., 2001
TetS R	AGGAGTATCTACAATATTTAC	
FloR817 F	AGGTGATTTTTGGTCCGCTCT	This study
FloR817 R	ATGTCGTCGAACTCTGCCAAA	
aadA2:510 F	TTGTTGGTTACTGTGGCCGT	This study
aadA2:510 R	CTGGGCAGGTAGGCGTTTTA	
TetM F	GTTAAATAGTGTTCTTGGAG	Aarestrup et al., 2000
TetM R	CTAAGATATGGCTCTAACAA	
TetE F	TCGGGATTGTTAGTTGTCTTTTTC	Fan et al., 2007
TetE R	GTGGATTACCCTACCTGGATGGA	
TetA F	GCTACATCCTGCTTGCCTTC	Dahshan et al., 2010
TetA R	CATAGATCGCCGTGAAGAGG	
Sul1F	CTGAACGATATCCAAGGATTYCC	Heuer and Smalla, 2007
Sul1R	AAAAATCCCATCCCCGGRTC	
Sul2 F	CTCAATGATATTCGCGGTTTYCC	
Sul2 R	AAAAACCCCATGCCGGGRTC	
StrB F	ATCGTCAAGGGATTGAAACC	Rosengren et al., 2009
StrB R	GGATCGTAGAACATATTGGC	
StrA F	CTTGGTGATAACGGCAATTC	
StrA R	CCAATCGCAGATAGAAGGC	

### 2.4 Biocompounds for bioassays

Biocompounds andrographolide (ANDR), rutin (RUT), quercetin (QUE), docosanol (DOC), lanosterol (LAN), and thymohydroquinone (THQ) were obtained from Sigma-Aldrich (St. Louis, MO, United States) and were prepared as corresponding stock solutions following product information: ANDR - 3mg/ml in DMSO; RUT 2.5 mg/ml using DMSO; QUE- 0.5 mg/ml in DMSO; DOC - 1mg/ml in ethanol; LAN - 1mg/ml in DMSO; THQ - 1mg/ml in DMSO. All stock solutions were stored at -20°C upon preparation and used immediately to ensure freshness and stability.

### 2.5 Antibacterial assay and determination of minimum inhibitory concentration

Disc diffusion method for antimicrobial susceptibility testing was carried out to assess the presence of antibacterial activities of biocompounds (Rezaei et al., 2011). The 24-hour culture of *Aeromonas* spp. in nutrient broth was adjusted to 0.5 McFarland standard. Sterile swabs were immersed into the bacterial culture and were aseptically swabbed onto the surface of bacterial medium plates evenly. Twenty μl of each treatment, positive control Enrofloxacin and distilled water for negative control, were pipetted on sterile petri plates containing sterile paper discs which were dipped for 30 minutes. The discs were placed equidistantly on the surface of the agar medium specific for each pathogen using sterile forceps. Treatments were done in triplicates and incubated at 37°C for 24 hours. The zone of inhibition was observed after 12 and 24 hours of incubation. Positive result in the antibacterial assay established the command to proceed on the determination of Minimum Inhibitory Concentration (MIC) determination. The determination of Minimal Inhibitory Concentrations (MICs in mg/ml) of the treatments were determined by performing an adaptation of the method according to Clinical and Laboratory Standards Institute (2015) and with an inoculum of 1 × 10^5^ Colony-Forming Unit/ml. Two-fold dilution method using Nutrient Broth was used to prepare a series of concentrations for the assay. The assay was carried out in a 96-well polystyrene microtiter plate, with 10 wells for each concentration. MIC is defined as the minimum concentration of the biocompounds which inhibit the visible growth of the *Aeromonas* sp. The lowest concentration that reduced the rezasurin dye was considered the MIC. In the case of THQ with observed antibacterial activity, the sub-MIC was through rezasurin-based assay in microtiter plates with two-fold dilution consisting of 8 concentrations – 1 mg/ml, 0.5 mg/ml, 0.25 mg/ml, 0.125 mg/ml, 0.0625 mg/ml, 0.03125 mg/ml, 0.0156 mg/ml and 0.0078 mg/ml (Gajic et al., 2022).

### 2.6 Microtiter plate biofilm formation assay

180 µl of overnight cultures of *Aeromonas* sp. were added to 20 µl biocompounds in each well and incubated at 30°C for 40 h. Untreated bacterial cultures served as negative control. A biological blank (PBS) was also prepared. The setup was run twice, with each run consisting of 6 replicates. After incubation, microtiter plates were rinsed with sterile distilled water five (5) times to remove planktonic cells, air-dried for 45 min, and stained with 150 µl of 1% crystal violet solution. Plates were rinsed five (5) times to remove excess stain. Quantification of biofilm production was done by adding 200 µl of 95% ethanol to destain the wells. Then, 100 μl from each well was transferred to a new microtiter plate and the optical density (OD) values were measured at 595 nm (MultiSkan FC, Thermo Scientific) (Fernando and Judan Cruz, 2020).

### 2.7 Expression analysis of ARGs and biofilm-linked genes

The total RNA of the bacteria were extracted using the RNeasy Minikit protocol (Qiagen, GmbH, Germany). For each sample, 25-50 mg acid-washed glass beads (150-600 μm diameter) were weighed in 2 ml safe-lock tubes. Bacteria were collected by centrifugation at 5000 x g for five minutes at 4°C. The supernatant was decanted and aspirated to ensure the removal of the remaining media. Buffer RLT was added (350 μl for <5 x 10^8^ and 700 μl for 5 x 10^8^ – 1 x 10^9^ number of bacteria). The suspension was transferred into the 2ml safe-lock tube containing the acid-washed beads. Cells were disrupted in the Tissue Lyser for five minutes at maximum speed. The suspension was centrifuged for 10 seconds at maximum speed. The supernatant was transferred into a new tube and the volume of the sample was determined. An equal volume of 70% ethanol was added and mixed by pipetting. Up to 700 μl lysate was transferred to a spin column placed in a 2 ml collection tube and centrifuged for 15 seconds at ≥8000 x g. The flow-through tube was discarded. 700 μl Buffer RW1 was added to the spin column. With the lid closed gently, the spin column was centrifuged for 15 seconds at ≥8000 x g to wash the spin column membrane. The spin-column was placed in a new 2 ml collection tube with the flow-through. Lids were closed gently and centrifuged at full speed for one minute. The spin-column was placed in a new 1.5 ml collection tube. 30-50 μl RNase-free water was added directly to the spin column membrane and was centrifuged for one minute at ≥8000 x g to elute the RNA (Qiagen, 2023).

The expression analysis of ARGs and biofilm-linked genes was done using qRT-PCR with specific primers (**Tables 1**, **2**) using Promega GoTaq ^®^ 1-step RT-qPCR kit following the recommended proportions. A 10 μl reaction mix was prepared for each of the samples. qRT-PCR conditions included the following: 1 cycle at 42°C for 5 min and 95°C for 2 min for initial denaturation, followed by 45 cycles at 95°C for 20 s for denaturation, 55°C for 20 s for annealing, and 72°C for 20 s for the extension. For the ARGs expression, annealing temperature ranged from 55°C to 60°C. The relative expression of genes was analyzed using the LIVAK method of 2^−ΔΔCt^ (Livak and Schmittgen, 2001).

**Table 2:** Primers used in qRT-PCR analysis of biofilm-linked genes.

Primer	Primer sequence (5’-3’)	References
ahyR -F	TTTACGGGTGACCTGATTGAG	Patel et al., 2017
ahyR -R	CCTGGATGTCCAACTACATCTT	
ahyI -F	GTCAGCTCCCACACGTCGTT	Dong et al., 2020
ahyI -R	GGGATGTGGAATCCCACCGT	
litR -F	CATCGAGGTGTTCTCCCGTC	Sun et al., 2021
litR -R	TCATCCACCAGCTCTTCACG	
casgAB -F	TTGTTTCTGGTGGATCTGGATTA	
casgAB -R	GGCATTGAGCAGCACGGTA	
fleQ -F	ACTTCCCACAGCAACTTCA	
fleQ -R	CCTTGTCGTGGGTCTGTTGA	
16S F	GCACAAGCGGTGGAGCATGTGG	
16S R	CGTGTGTAGCCCTGGTCGTA	

### 2.8 Statistical analysis

To determine statistical differences in biofilm formation, quantification through OD values were analyzed through an independent sample non-parametric Mann-Whitney U test with 0.05 level of significance using SPSS 13.0 program. The statistical analyses for the gene regulation were done using the Kruskal-Wallis test (non-parametric ANOVA) where means between the control and experimental setup were compared and the significance were determined if the F-values were greater than the F-crit at 0.05 level of significance. For the analysis of the relative mRNA expression in qPCR, the LIVAK method (2−ΔΔCt) was used. The correlation analyses between biofilm formation and biofilm-linked genes, biofilm formation and ARGs as well as biofilm-linked genes and ARGS were done through Spearman correlation coefficient using the function pairs*.panel* in *psychs* package in R v. 4.3.1 following the standard procedure for non-parametric correlation analysis.

## 3 Results

### 3.1 16S rRNA Identification of *Aeromonas* wastewater bacteria

Comparison of 16S rRNA sequences of 60 wastewater bacterial isolates with the NCBI database (BLAST) confirmed the presence of known bacterial pathogens, from which five (5) species of *Aeromonas* were selected for further experiments on the detection and inhibition of ARGs and biofilm formation. These are *Aeromonas veronii, Aeromonas jandaei, Aeromonas hydrophila, Aeromonas dhakensis* and *Aeromonas caviae*.

### 3.2 Detection of ARGs

Major ARGs coding resistance to streptomycin, tetracyclines, sulfonamide and aminoglycosides were evaluated for their occurrence in the identified wastewater *Aeromonas* species. To identify and analyze the presence of ARGs within the *Aeromonas* spp., sequences were checked on ResFinder 4.5.0 - Center for Genomic Epidemiology (CGE) (Clausen et al., 2018; Bortolaia et al., 2020). Eleven ARGs were evaluated for their presence: *StrA, StrB, TetS, TetM, TetE, TetA, FloR, Sul1, Sul2, aadA1* and *aadA2*. The results obtained from ResFinder revealed the presence of three ARGs in two *Aeromonas* spp: *A. caviae* carries three ARGs namely *aadA1*, *aadA2* and *sul1; sul1* was also detected in *A. jandaei.*

### 3.3 Biocompounds inhibit the expression of *Aeromonas* ARGs

**Figure 1:**
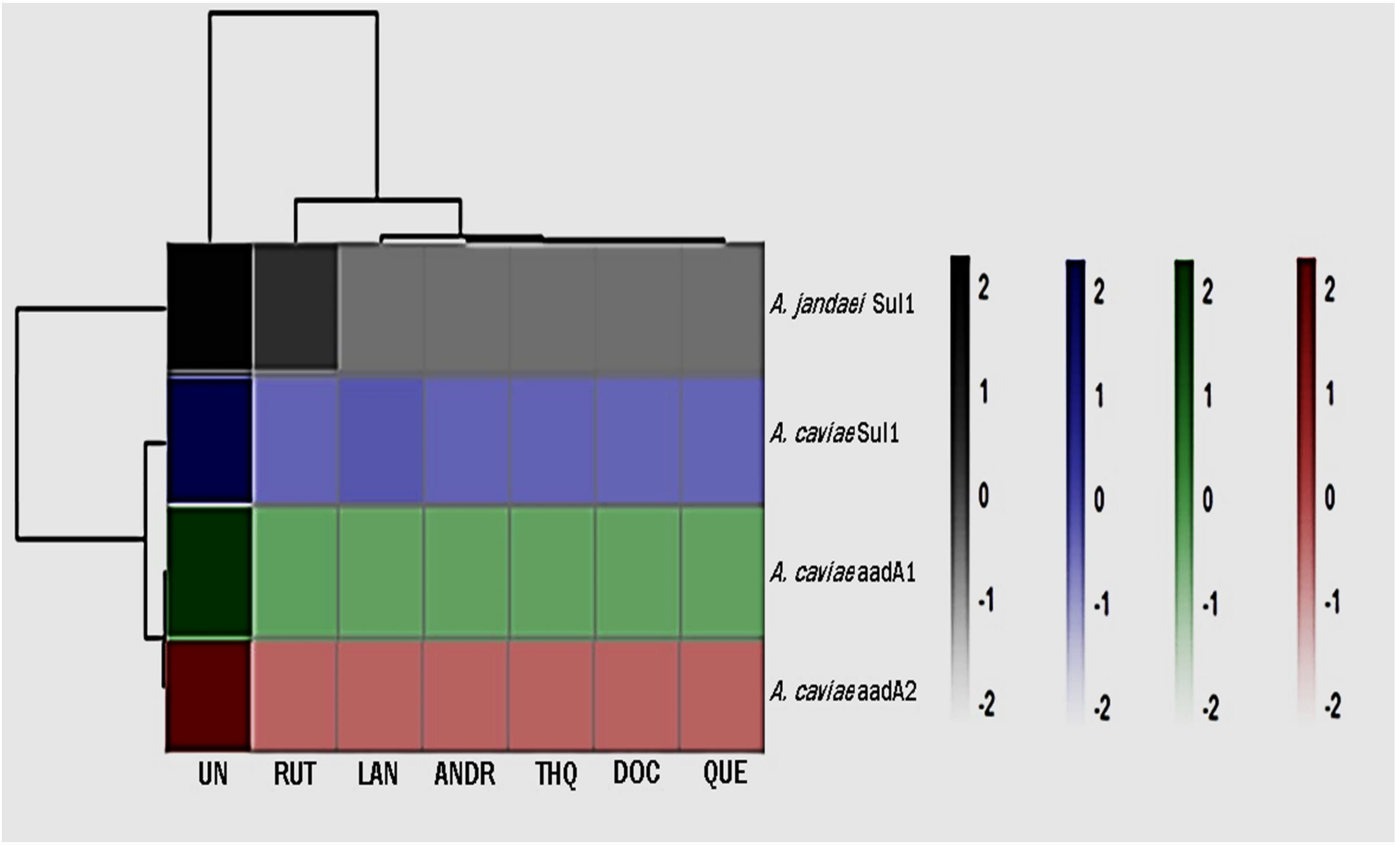
Heatmap of gene expression of ARGs as affected by the treatments. UN (untreated); RUT (rutin); LAN (lanosterol); QUE (quercetin); ANDR (andrographolide); DOC (docosanol); THQ.

The changes in gene expression levels were visualized using a heatmap to present effect of the biocompounds on the expression of ARGs (**Figure 1**; **Supplementary Table 1**). The numbers on the right side of the heat map indicate the standardized relative quantification values. The cluster delineating between the effect of the biocompounds versus the untreated sample is clear and distinct. The decrease in *aadA1*, *aadA2* and *sul1*expression was statistically significant (*p <0.05*) indicating a downregulating effect of the biocompounds. While RUT’s effects on ARG expression varied slightly from the other biocompounds, still, showed substantial downregulation as compared to the untreated (UN) (*p <0.05*).

### 3.4 Antibacterial activity of biocompounds against the *Aeromonas* spp.

Before conducting evaluations on biofilm activity, an antibacterial screening was performed to ensure that any observed reduction in the production of biofilm was not due to antibacterial properties. For biocompounds with confirmed antibacterial activity as noted by a zone of inhibition, the sub MIC was determined through a rezasurin-based assay. The biocompounds ANDR, RUT, QUE, DOC and LAN did not show any antibacterial activity in the disk diffusion assay across all tested *Aeromonas* spp, hence, their original concentrations were retained for further assessments. THQ demonstrated antibacterial activity against all Aeromonas spp. except *A. veronii*, hence, the rezasurin-based assay was performed to determine the sub-MIC that resulted to 0.5 mg/ml. This sub-MIC was then used for both assays on ARGs and biofilm since the objective was to see the effects on both mechanisms using the same concentration.

### 3.5 Biofilm formation inhibitory effect of the biocompounds against *Aeromonas* spp.

**Figure 2:**
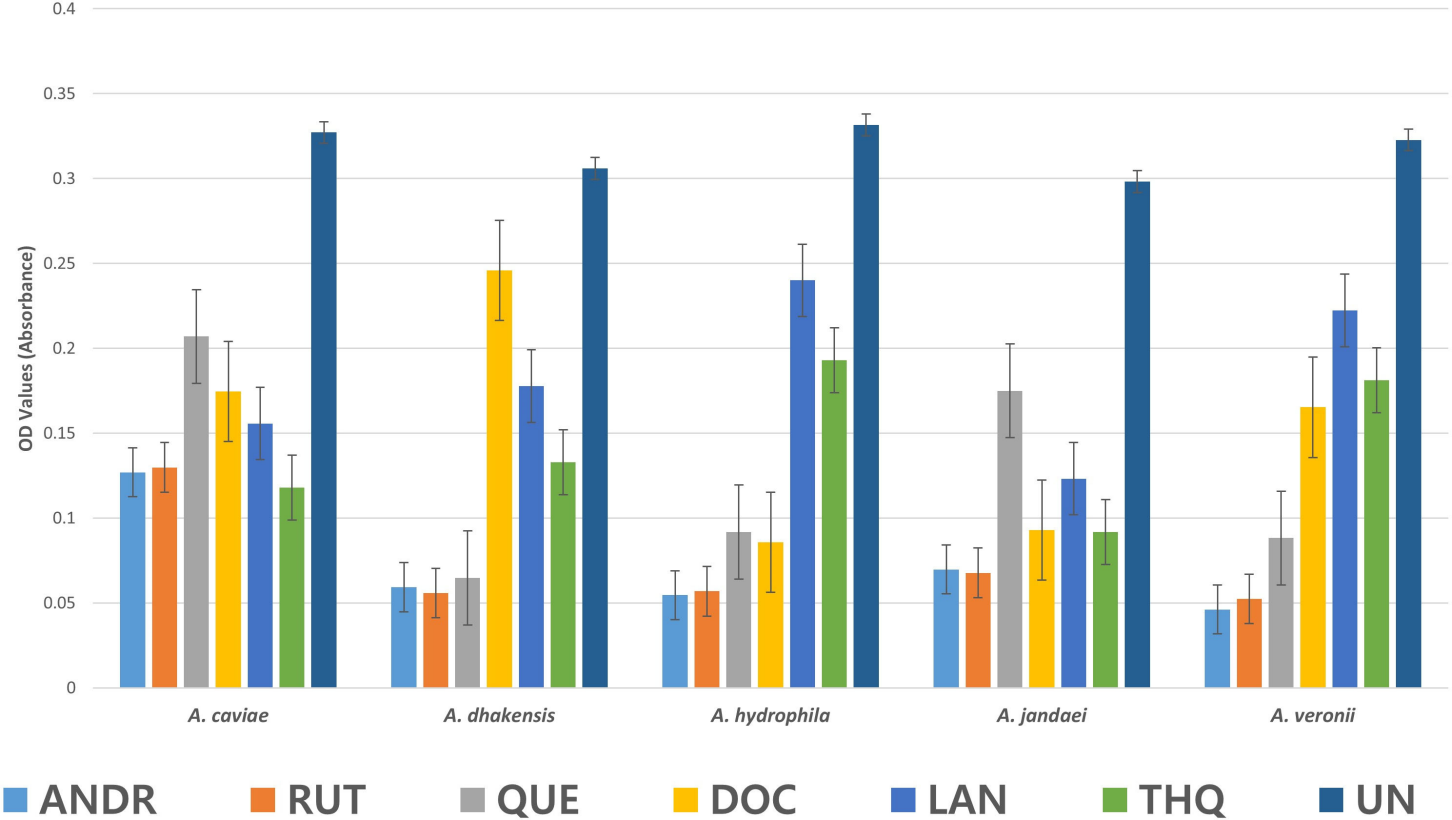
Mean OD values in *Aeromonas* sp. as affected by the biocompounds.

The biofilm formation activity in *Aeromonas* spp as affected by the biocompounds was lower than the control (UN) (**Figure 2**). The values presented are OD values for each treatment. In *A. caviae* strong inhibition by ANDR (0.127), RUT (0.129), DOC (0.174), LAN (0.156) and THQ (0.118) were noted compared to (UN) (0.327) (*p < 0.01*). *A. caviae* treated with QUE was not significantly different but showed lower OD values at 0.207 compared to UN (*p > 0.05*). The OD values in *A. dhakensis* also significantly decreased (*p < 0.01*) when treated with AND (0.059), RUT (0.056), QUE (0.65) and LAN (0.178). DOC (0.246) and THQ (0.133) showed lower OD values but was not significantly different from the UN (control) (0.306) (*p > 0.05*). The same significant observations were seen in *A. hydrophila* when exposed to ANDR (0.055), RUT (0.057), QUE (0.092) and DOC (0.086) (*p < 0.01*). Although LAN (0.240) and THQ (0.193) showed decrease in biofilm formation, it showed no significant difference with UN (0.33) (*p > 0.05*); in *A. jandaei*, all compounds except QUE (0.175) have strong anti-biofilm activity versus UN (0.298) (*p < 0.01*) as ANDR (0.069), RUT (0.068), docosanol (0.093), lanosterol (0.123) and THQ (0.092; and in *A. veronii*, ANDR (0.046), rutin (0.052), quercetin (0.088), docosanol (0.165) substantially suppressed biofilm formation (*p < 0.01*); OD values of LAN (0.222) and THQ (0.181) was observed lower but not significant compared to UN (0.323) (*p > 0.05*). The mean OD values in *Aeromonas* spp. treated with the biocompounds showed significant decrease in biofilm formation indicates efficiency in controlling the growth of biofilm. Overall, all biocompounds showed inhibition in biofilm formation against the wastewater *Aeromonas* spp.

### 3.6 Biocompounds inhibit the expression of *Aeromonas* biofilm-linked genes

**Figure 3:**
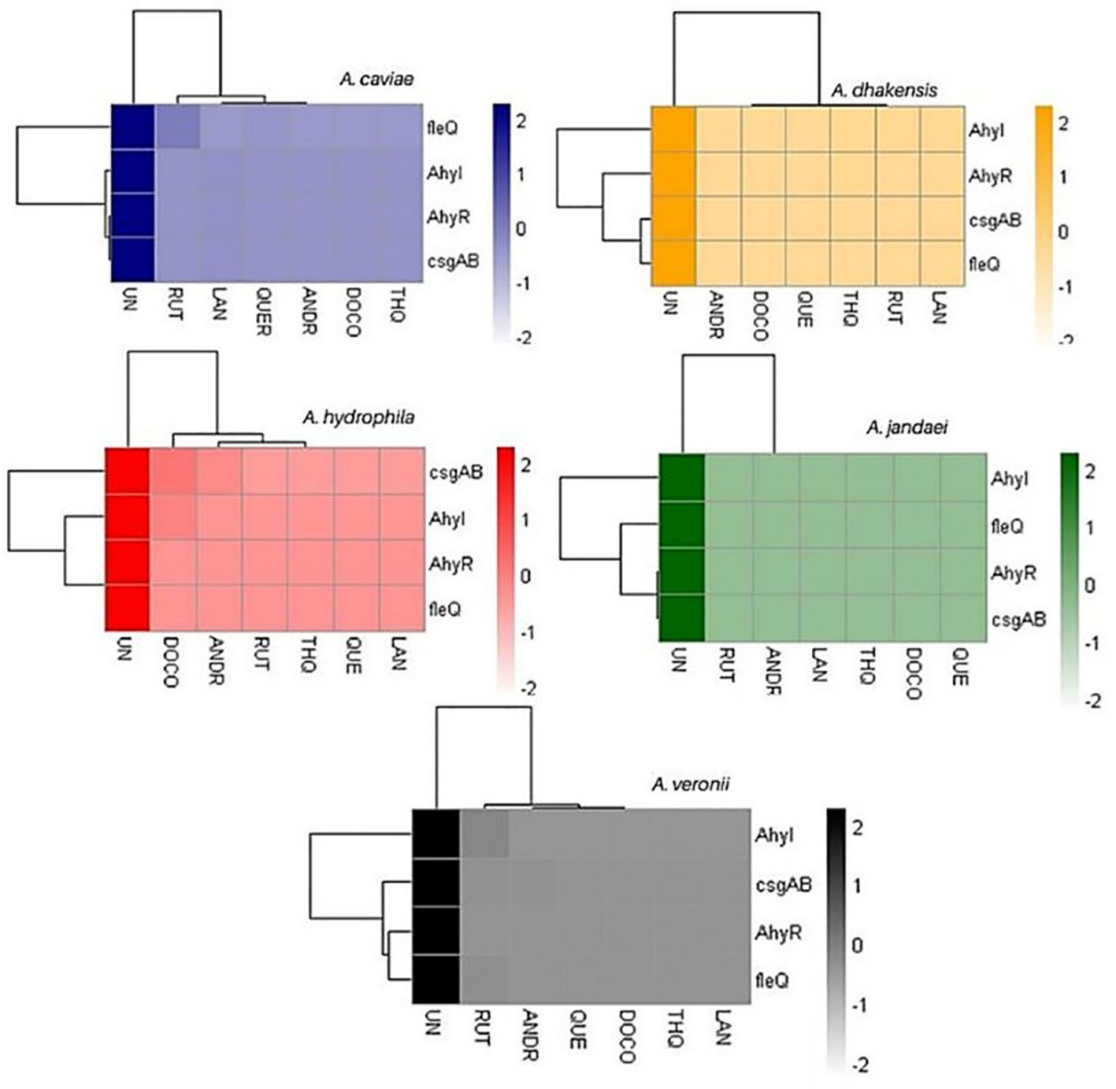
Heatmap of gene expression of biofilm-linked genes as affected by the treatments. UN (untreated); RUT (rutin); LAN (lanosterol); QUE (quercetin); ANDR (andrographolide); DOC (docosanol); THQ (thymohydroquinone).

The changes in gene expression levels were visualized in a heatmap, indicating the relative expression levels of key biofilm-linked genes across different samples. The heatmap of relative gene expression of biofilm-linked genes as affected by the compounds clearly shows defined clustering (**Figure 3**; **Supplementary Table 2**). The biocompounds ANDR, QUE, RUT, DOC, LAN and THQ clustered in one group showing similar downregulation of all genes compared to the untreated culture (UN) which suggests significant effects (*p <*0.05) of these compounds against the expression of the genes. This can be noted in *A. dhakensis* and *A. jandaei* (*p <*0.05). Different effects were observed in *A. caviae*, *A. hydrophila*, and *A. veronii*. For instance, fleQ as affected by RUT (*A. caviae*), *AhyI, AhyR, fleQ*, and *casgAB* in ANDR (*A. hydrophila* and *A. veronii*), DOC (*A. hydrophila*), RUT (*A. veronii*) showed higher expression of genes than other compounds, nevertheless, still showed significant downregulation when compared to treatments without the compounds (*p <*0.05).

### 3.7 Correlation analysis

**Figure 4:**
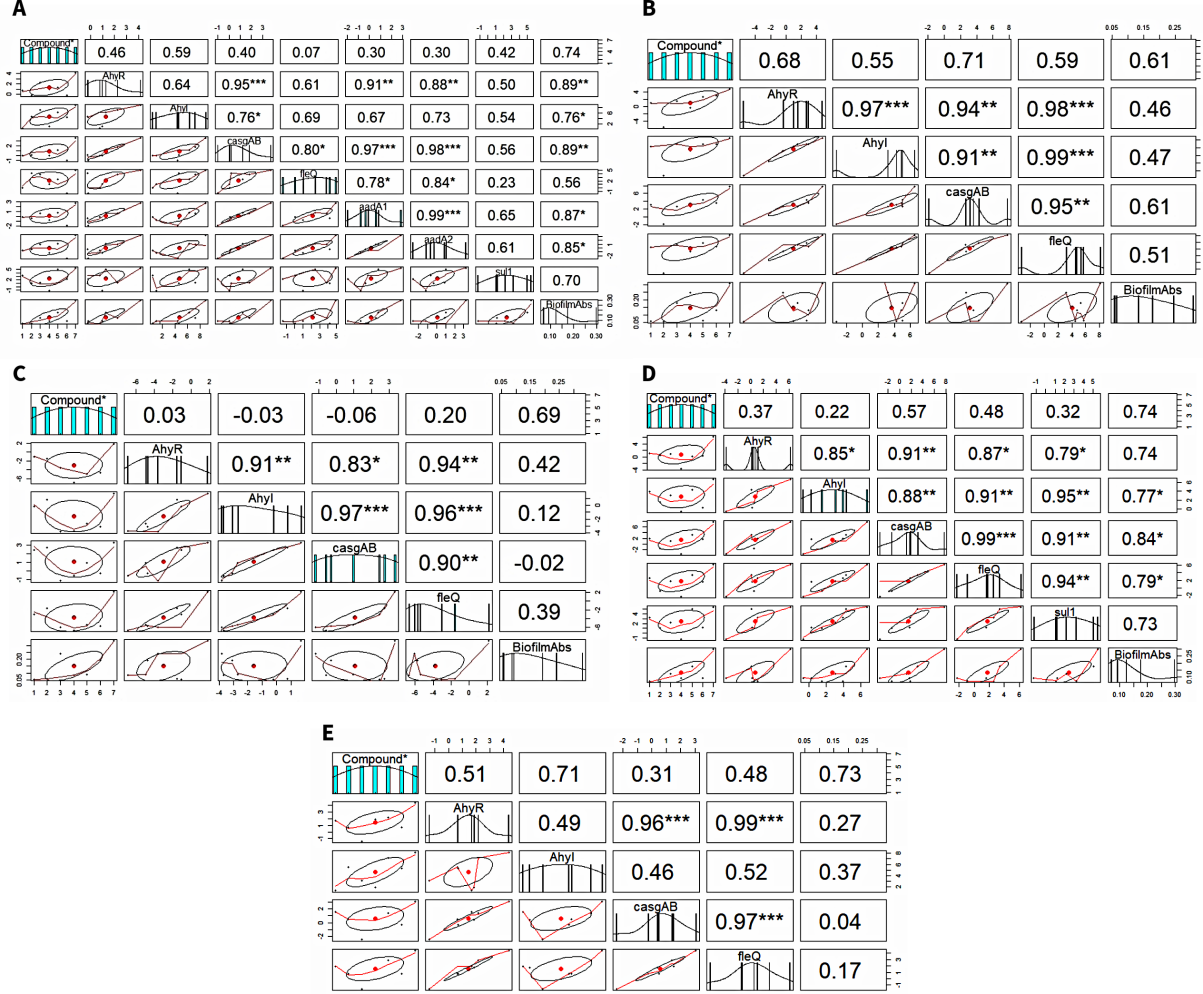
Correlation analysis between biofilm formation, biofilm-linked genes and ARGs. **(A)** *A. caviae*, **(B)** *A. dhakensis*, **(C)** *A. hydrophila*, **(D)** *A. jandaei*, **(E)** *A. veronii*.

The Spearman’s rank correlation coefficient (R) and its significance level (p-value) were calculated to analyze the relationship between the expression levels of genes and biofilm formation (**Figure 4**). In *A. caviae*, a strong significant positive correlation between biofilm-linked gene expression and biofilm formation was noted: *AhyR* (R = 0.89; *p* < 0.01), *AhyI* (R = 0.76; *p* < 0.05) and *casgAB* (R = 0.89; *p* < 0.01). This was also observed between biofilm formation and expression of ARGs *aadA1* (R = 0.87; *p* < 0.05) and *aadA2* (R = 0.87; *p* < 0.05). A strong positive correlation was also noted between the *aadA1* and biofilm-linked genes *AhyR* (R = 0.91; *p* < 0.01), *casgAB* (R = 0.97; *p* < 0.001) and *fleQ* (R = 0.78; *p* < 0.05), as well as in *aadA2*: *AhyR* (R = 0.88*; p* < 0.01), *casgAB* (R = 0.98; *p < 0.001*) and *fleQ* (R = 0.84; *p* < 0.05. *A* strong significant positive correlation between biofilm-linked gene expression and biofilm formation was also seen in *A. jandaei*: *AhyI* (R = 0.77; *p* < 0.05), *casgAB* (R = 0.84; *p* < 0.05), *fleQ* (R = 0.79; *p* < 0.05). sul1 expression registered high significant correlation values between all biofilm-linked genes *AhyR* (R = 0.79; *p* < 0.01), *AhyI* (R = 0.95; *p* < 0.01), *casgAB* (R = 0.91; *p* < 0.01) and *fleQ* (R = 0.94; *p* < 0.01). The R value between biofilm formation and sul1 expression (0.73) was not significant, nevertheless, still showed a strong correlation. No ARGs were detected in *A. dhakensis*, *A. hydrophila* and *A. veronii*, hence, the correlation only presented biofilm and biofilm-linked genes. R values displayed positive correlation between biofilm formation and biofilm-linked genes in *A. dhakensis*, *A. hydrophila* and *A. veronii* through the R values but were not significant. However, strong interactions (*p* < 0.001) were observed between biofilm-linked genes.

## 4 Discussion

In this study, three ARGs—aminoglycoside resistance genes *aadA1* and *aadA2*, and sulfonamide resistance gene *sul1*—were identified in wastewater *Aeromonas* spp. This work shows effective downregulation of these ARGs when exposed to the biocompounds, suggesting their potential in targeting AMR. These ARGs are involved in enzymatic degradation or modification of respective antibiotics i.e., *sul1* encoding the DHPS enzymes with pronounced insensitivity to sulfonamides (Venkatesan et al., 2023); *aadA1* and *aadA2* modify aminoglycosides by adding an adenyl group rendering them inactive. As such, their efficiency adds up to strategies targeting antimicrobial-resistant enzymes and opens up the possibility of potentially developing these compounds for use in combination therapies as antibiotic adjuvants. Bacteria employ various mechanisms to resist antibiotics, including efflux pumps, antibiotic structural modifications, and in enzyme-catalyzed deactivation (El-Khoury et al., 2022; Murugaiyan et al., 2022). Targeting these mechanisms is crucial for enhancing antibiotic efficacy. Antibiotic adjuvants are used in cases of combination therapies (Kumar and Tudu, 2023) to augment the action of antibiotics by suppressing a mechanism of resistance thereby increasing bacterial susceptibility (Álvarez-Martínez et al., 2020). Natural products, for example, extracts from *Rhus coriaria* (Adwan et al., 2010) and plant-based compounds like tea catechins (Hu et al., 2002), quercetin and kaempferol (Lin et al., 2008), have shown synergy with antibiotics against resistant pathogens, such as *Pseudomonas aeruginosa* and MRSA (methicillin-resistant *Staphylococcus aureus*). While adjuvants are less effective in monotherapy, their combination with traditional antibiotics enhances therapeutic outcomes (Álvarez-Martínez et al., 2020). Efflux pump inhibitors (EPIs), a class of antibiotic adjuvants, have shown promise in preclinical and clinical settings (Van Bambeke et al., 2006; Zhang et al., 2023; Compagne et al., 2023) though challenges remain in their development, including pharmacokinetic profiling and addressing bacterial specificity, particularly their potential side effects on beneficial bacteria. Combination therapy of antibiotics + natural product adjuvants offer new promising strategies in synergistic approaches to manage recurring infections and control the spread of AMR.

The *Aeromonas* spp. identified in activated sludge in this study aligns with the previous reports of their consistent presence and ubiquity in wastewater samples (Skwor et al., 2020). *Aeromonas* spp are key indicator bacteria for assessing water quality risks, chiefly in antimicrobial resistance and health issues related to aquatic environments (Usui et al., 2016). The presence of the detected ARGs likely reflects past exposure and selective pressure from antibiotics, notably aminoglycosides and sulfonamides, which are frequently detected in wastewater before and after treatment (Antunes et al., 2005). Sulfonamides are widely disseminated in wastewater, often exceeding sulfonamide levels in aquatic environments linked to wastewater discharge (Proia et al., 2016; Hanna et al., 2018; Kovalakova et al., 2020). In contrast, aminoglycosides, like streptomycin, are typically found at lower concentrations, possibly due to reduced clinical use attributed to toxicity effects and has been in applied only in veterinary practices (Mutuku et al., 2022). *Aeromonas* spp. exhibits broad antibiotic resistance, notably to β-lactams, quinolones, aminoglycosides, sulfonamides, and tetracyclines, mediated by ARGs on mobile genetic elements (MGEs) such as plasmids, insertion sequences, transposons, and mobile integron gene cassettes (Piotrowska and Popowska, 2015). In *Aeromonas*, *aadA1* and *aadA2* are the most predominant ARGs in resistance integrons (Partridge et al., 2009; Piotrowska and Popowska, 2014; Khorshidi et al., 2022) while *sul*1 is considered a good marker of Class 1 integrons (Arabi et al., 2015; De los Santos et al., 2021). As also seen in this work, *sul1* and *aadA* are found to be colocalized in *Aeromonas* species (Lee et al., 2023) which provides higher risk of transmission and renders the *Aeromonas* spp co-resistance to wider spectrum of antibiotics. Although this study only detected three out of 11 target ARGs, their detection in two isolated *Aeromonas* species in WWTPs already provides evidence not only of their presence but also shows their diversity of AMR mechanisms (Piotrowska and Popowska, 2014).

Biofilm formation in *Aeromonas* spp was also effectively inhibited by all biocompounds evaluated in this study (**Figure 2**). When comparing the OD values, the treatment without biocompounds showed considerably higher values, indicating higher biofilm formation. Biofilm is a critical factor in bacterial virulence and the evolution of AMR, directly contributing to approximately 80% of human microbial infections (Shunmugaperumal, 2010; Davies, 2003). Our findings demonstrate that the evaluated biocompounds disrupt biofilm formation, potentially through interference with quorum sensing (QS). QS is a communication mechanism that bacteria use to coordinate biofilm formation by influencing adhesion and exopolysaccharide (EPS) production (Miller and Bassler, 2001; Shrout and Nerenberg, 2012). By targeting QS, the biocompounds in this work may reduce pathogenicity without affecting bacterial growth, thereby lowering selective pressure and the development of antimicrobial resistance. This alternative therapeutic approach of inhibiting QS and biofilm formation offers a promising strategy for managing bacterial infections and combating AMR.

By interfering with QS cell-cell signaling, biofilm synthesis genetic pathways, adhesins, efflux pumps, and motility by repressing flagellar genes, phytochemicals function as natural antibiofilm agents that can target all stages of development (Mishra et al., 2020). Corollary to the inhibition of biofilm formation, biofilm-linked genes *ahyR*, *ahyI*, *fleQ* and *casgAB* were subsequently downregulated confirming the effects of the biocompounds and likewise points out that the compounds were able to target these genetic pathways. AhyR and AhyI are homologues of the Lux QS proteins (Kirke et al., 2004). Biofilm development in *Aeromonas* is dependent on the production of many QS signaling molecules, *N*-acylhomoserine lactone (AHL), via the AhyRI pathways (Lynch et al., 2002; Jin et al., 2020) wherein *ahyR* encodes AhyR which acts as a transcription regulator of *ahyI* for the synthesis of AHLs. *fleQ* is involved in the regulation of flagellar biogenesis and motility which is closely intertwined with the processes leading to biofilm formation (Du et al., 2020; Laganenka et al., 2020; Benyoussef et al., 2022). *csgAB* operon is responsible for the production of curli fimbriae which facilitates cell adhesion and biofilm formation (Barnhart and Chapman, 2006; Kozlova et al., 2011; Boya et al., 2022).

Our study evaluated the efficacy of six biocompounds in inhibiting biofilm formation and ARG expression in *Aeromonas* spp. The results demonstrated significant antibiofilm activity for all tested compounds, with andrographolide consistently showing substantial inhibition. Quercetin also showed significant inhibition in the majority of cases but was not as effective in all *Aeromonas* spp. tested. Andrographolide, a diterpenoid from *A. paniculata*, effectively reduced biofilm formation in *Aeromonas*, corroborating its known antimicrobial properties against high-risk pathogens such as *S. aureus*, *Escherichia coli*, and *P. aeruginosa* (Guo et al., 2014; Banerjee et al., 2017; Zhang et al., 2020). Andrographolide has also been reported to inhibit QS in *Listeria monocytogenes* by targeting the *Agr* system (Yu et al., 2022). Quercetin, a flavonoid, suppressed QS molecules and biofilm-related genes in *Aeromonas*, aligning with its previously reported effects on *P. aeruginosa* and *Klebsiella pneumoniae*, where it inhibited QS molecules *lasI, lasR, rhlI*, and *rhlR*, reducing pyocyanin production and swarming (Gopu et al., 2015; Ouyang et al., 2016; Paczkowski et al., 2017; Wang et al., 2021). It also affected gene expression linked to flagellar motility, biofilm formation, and virulence in *Vibrio parahaemolyticus* (Roy et al., 2022). Docosanol, primarily recognized for its antiviral properties (Katz et al., 1991; Pope et al., 1998; Sadowski et al., 2021), also exhibited antibiofilm activity in our study. This finding is consistent with limited reports on its bacterial effects, such as the suppression of biofilm and virulence factors in MRSA and the downregulation of stress response proteins (Lakshmi et al., 2020, 2022), and in *K. pneumoniae* (Umaru et al., 2019). Similarly, rutin has shown effectiveness against QS, biofilm formation, and expression of virulence genes in *E. coli* and *S. aureus* (Peng et al., 2018; Matilla-Cuenca et al., 2020). lanosterol and thymohydroquinone (THQ), despite their limited prior research on antimicrobial properties, showed promising results in our assays. Lanosterol has documented anticancer (Ivankovic et al., 2006) and antioxidant attributes (Tesarova et al., 2011), while THQ has shown potential but remains largely unexplored in terms of antimicrobial properties. Natural products display a rich repertoire of chemically and structurally diverse molecules with novel antimicrobial activities. This diversity can target different aspects of bacterial mechanisms and ensures that bacteria cannot easily predict or develop resistance to phytochemicals simultaneously. The dynamic nature of these compounds allows them to interfere with various bacterial processes, making them promising candidates for combating biofilm-related infections and reducing the evolution of antimicrobial resistance. Our findings provide new insights into the antibiofilm and QS-inhibiting properties of these natural biocompounds in *Aeromonas* spp., suggesting their potential as alternative therapeutic agents to combat biofilm-related infections and reduce the evolution of antimicrobial resistance. This study contributes to the limited research on the effects of these compounds on biofilm formation and ARG expression, highlighting their promise as antibiotic adjuvants.

The association between the ARGs, biofilm-linked genes and biofilm formation within the *Aeromonas* species as affected by the compounds was explored. The strong positive correlations identified in our study suggest a clear interaction between biofilm formation and the expression of biofilm-linked genes along with ARGs. Our findings align with previous studies suggesting a link between antibiotic resistance and biofilm formation, reinforcing that biofilms play a crucial role in the persistence and spread of antibiotic-resistant strains. The correlation between ARGs and biofilm-linked genes somehow suggests the coordinated regulation of these genes in the development of biofilm-associated antibiotic resistance. To strengthen these findings, future studies could consider increasing the sample size or conducting relevant experiments to further elucidate these correlations. This association has significant implications for simultaneously managing two resistance mechanisms, as well as understanding bacterial resistance mechanisms in biofilms. ARGs, such as in efflux pumps and antibiotic-modifying enzymes, have been linked to AMR in bacteria in biofilms (Kvist et al., 2008; Soto, 2013) wherein their absence can lead to diminished biofilm formation due to compromised adherence of microorganisms and the establishment of mature biofilm structures (Ren et al., 2024), thus, ARGs are now an emerging target for anti-biofilm applications and in enhancing the efficacy of AMR strategies. The limitations of this study confined the number of ARGs that could be detected to only 11, and given that only three ARGs were found and none were efflux pumps, it is plausible that other efflux pumps associated with biofilm formation in *Aeromonas* spp. were also affected. Hence, given this limitation, it is evident that further detection of other ARGs should be done, particularly those associated with efflux pumps. This will enhance the evaluations on the full spectrum of AMR mechanisms influenced by the biocompounds.

Suppression of both ARGs and biofilm formation by the biocompounds point to their significant role in the future of wastewater treatment. Since WWTPs are recognized as hotspots for horizontal gene transfer (HGT) of ARGs between bacterial populations, targeting these mechanisms in wastewater can prevent the transmission of antibiotic resistance, a critical issue concerning wastewater treatment. By reducing the transmission of ARGs in wastewater, there is a decreased risk of antibiotic resistance spreading into the environment, which could affect human health and ecosystems (Che et al., 2019). This also addresses reduction of biofouling in bioreactors. Reduction in biofouling, is a constant concern in wastewater treatment, and this justifies the application of biocompounds substances as a cost-effective and environmentally friendly solution paving the way for sustainable solutions to wastewater treatments and its implication to global health.

## 5 Conclusion

To combat the increasing multidrug resistance in *Aeromonas*, a comprehensive, multifaceted approach is essential, targeting both ARGs and biofilm formation. This work demonstrates that naturally-derived compounds effectively address these issues. Specifically, our results revealed that these compounds significantly reduced ARG expression levels and markedly decreased biofilm formation in *Aeromonas.*

The study highlights the effectiveness of these natural products in mitigating the development and transmission of AMR in wastewater bacteria. By disrupting biofilm formation and inhibiting ARG expression, these compounds present a promising strategy for enhancing sustainable wastewater treatment and preventing biofouling in treatment facilities. Furthermore, the ability of these biocompounds to act as adjuvants to antibiotics — by inhibiting ARGs and biofilms — offers a significant advancement in improving the efficacy of antibiotic therapies for biofilm-forming pathogens.

Overall, the findings underscore the potential of these natural products to provide a dual-action approach against AMR, both in molecular and structural aspects of bacterial resistance. This work not only addresses the challenge of AMR in wastewater bacteria but also contributes to reducing the broader burden of AMR. The study emphasizes the need for continued exploration of these bioactive compounds to fully understand their mechanisms and maximize their potential in addressing the global AMR crisis.

## Data Availability

All relevant data is contained within the article: The original contributions presented in the study are included in the article/**Supplementary Material**, further inquiries can be directed to the corresponding author/s.
