# Supplementary material for: Natural compound-induced downregulation of antimicrobial resistance and biofilm-linked genes in wastewater Aeromonas species
Source: Front Cell Infect Microbiol. 2024 Oct 14;14:1456700. doi: 10.3389/fcimb.2024.1456700 (PMC11513397; doi:10.3389/fcimb.2024.1456700)
Supplement: Supplementary file 1 [file Table1.docx]

Supplementary Table 1. Log values of detected ARG mRNA expression values in *Aeromonas s*pecies treated with the biocompounds.

|  | ANDRO | DOCO | RUT | QUER | LAN | THQ | UN |
| --- | --- | --- | --- | --- | --- | --- | --- |
| A. caviae Sul1 | 1.593533 | 0.924996 | 13.43765 | 0.28204 | 47.71272 | 3.348951 | 878.3664 |
| A. caviae aadA1 | 0.7643 | 0.443414 | 6.606212 | 0.162671 | 3.826172 | 0.011065 | 700.9467 |
| A. caviae aadA2 | 0.219179 | 0.479944 | 11.2762 | 0.064165 | 7.809191 | 0.002293 | 1785.826 |
| A. jandaei Sul1 | 1658.148 | 11.69472 | 108096.2 | 0.060303 | 16.07585 | 158.7979 | 302386.1 |
