# Supplementary material for: Natural compound-induced downregulation of antimicrobial resistance and biofilm-linked genes in wastewater Aeromonas species
Source: Front Cell Infect Microbiol. 2024 Oct 14;14:1456700. doi: 10.3389/fcimb.2024.1456700 (PMC11513397; doi:10.3389/fcimb.2024.1456700)
Supplement: Supplementary file 2 [file Table2.docx]

Supplementary Table 2. Log values of biofilm-linked mRNA expression values in *Aeromonas s*pecies treated with the biocompounds.

|  | ANDR | DOCO | RUT | QUE | THQ | LAN | UN |
| --- | --- | --- | --- | --- | --- | --- | --- |
| Acav AhyR | 6.07158799 | 0.563434955 | 18.927565 | 9.28307669 | 0.59971525 | 173.42689 | 15404.412 |
| Adhak AhyR | 9.86640872 | 3.57E-06 | 520.113519 | 0.42960313 | 850.010777 | 35.264644 | 54596.5223 |
| Ahydro AhyR | 0.08013948 | 0.028054216 | 1.25E-05 | 1.39E-07 | 2.06E-05 | 0.0002496 | 57.0639343 |
| Ajand AhyR | 13.4854647 | 3.429849067 | 1.39796993 | 0.00017737 | 1.9203267 | 6.7120663 | 1339565.16 |
| Avero AhyR | 48.1258648 | 4.466013594 | 150.027544 | 73.5814246 | 4.75358587 | 0.03761 | 23450.7282 |
| Acav AhyI | 2.61685723 | 42337.78131 | 1966163.49 | 10.0220487 | 24986.6049 | 18084315 | 860174725 |
| Adhak AhyI | 18465.2484 | 0.000514106 | 97465.1933 | 1431.2466 | 70840.4737 | 95475.596 | 3609138.59 |
| Ahydro AhyI | 0.61433382 | 5.983659567 | 0.00205821 | 0.00019795 | 0.00083387 | 0.0001484 | 52.3457318 |
| Ajand AhyI | 8500.50793 | 33.02666171 | 20358.3544 | 0.23717915 | 1.9203267 | 1244.7894 | 5198259.57 |
| Avero AhyI | 20.74227 | 335586.3975 | 15584607.9 | 79.4388161 | 198053.947 | 1109.8244 | 154394462 |
| Acav casgAB | 1.15495386 | 1.486913761 | 16.8633321 | 1.10611313 | 0.09400409 | 63.572139 | 5556.86124 |
| Adhak casgAB | 1501.85004 | 0.012211237 | 19758.9572 | 453.991815 | 3771.01319 | 407.35719 | 64153849.8 |
| Ahydro casgAB | 280.316624 | 542.7621859 | 9.17424384 | 0.25791094 | 0.48931031 | 0.064788 | 2125.12698 |
| Ajand casgAB | 74.238067 | 0.041172866 | 1419.7915 | 0.00038613 | 55.3578915 | 15.030146 | 50976653.6 |
| Avero casgAB | 33.447344 | 3.161698384 | 25.9466493 | 2.42146969 | 0.55374803 | 0.0034231 | 1202.62537 |
| Acav fleQ | 1.15344846 | 1.146976008 | 15311.4254 | 297.30867 | 10.5203117 | 0.0381775 | 84113.7771 |
| Adhak fleQ | 30415.6255 | 0.000500048 | 497325.772 | 1474.17583 | 52801.4241 | 195438.71 | 125654607 |
| AhydrofleQ | 0.0008543 | 0.025755567 | 3.77E-06 | 2.16E-07 | 1.92E-06 | 8.75E-07 | 147.028424 |
| Ajand fleQ | 380.917124 | 0.131957485 | 2492.4232 | 0.00491805 | 40.3608884 | 58.814493 | 1637806.32 |
| Avero fleQ | 90.5763289 | 6.357863917 | 748.497286 | 51.2374056 | 5.47710508 | 0.023529 | 26351.7887 |
